# Rupture of the Choroid, with Report of a Case

**Published:** 1901-11

**Authors:** Dunbar Roy

**Affiliations:** Clinical Professor of Eye, Ear, Nose, and Throat Diseases in the Atlanta College of Physicians and Surgeons, Atlanta, Ga.


### RUPTURE OF THE CHOROID, WITH REPORT OF A CASE.

BY DUNBAR ROY, M. D.,

Clinical Professor of Eye, Ear, Nose, and Throat Diseases in the Atlanta College of Physicians and Surgeons, Atlanta, Ga.

To the ophthalmologist cases of choroidal rupture are always of interest, from the fact that no two ever show the same clinical picture and in the majority of cases never exhibit the same etiology.

The following case is presented :

J. P., colored, age 26, presented himself at my office July 3d, 1897, with the following history : On the 26th of the previous month while traveling on the train he became involved in an altercation with the conductor, who evidently did not appreciate his gab, and thereupon struck him on the left eye and face with his fist. The patient claimed that the conductor used brass knucks, an idea probably evolved from the intensity of the blow. As a result of this the left eye and its adnexa became so swollen that the lids could not be opened. Cold cloths were used, nothing else being done up to the time the patient came to my office.

*Examination.*—Right eye appears normal. Left eye shows slight swelling of the lids and some redness of the bulbar conjunctiva. Pupil appears normal, but responds feebly to light and accommodation. Patient complains of no pain. Being hurried at this hour a minute ophthalmoscopic examination was not made. Vision seemed diminished. Patient was given a solution of atropin to use in the eye. Tension was normal.

One week later the patient returned. There was now very little congestion about the eye. Pupil fully dilated and regular. Vision equals 10-200. The perimeter shows marked contraction of the visual field for all colors. On ophthalmoscopic examination three crescentic ruptures could be seen in the choroid on the nasal side of the optic disk, distant about the width of the disk. The lowest was the largest, the other two diminishing towards the top. No hemorrhages could be seen. Patient complained of no pain. The same treatment was continued.

One week later the ruptures were still better defined. No congestion of the eye. Pupil well dilated. Some floating bodies could be seen in the vitreous. Vision the same. The patient was placed on the iodide of potassium internally. At the end of another week the ruptures could be seen even more distinctly. The vitreous opacities had somewhat decreased. Vision equalled 10-40. From this time on there were no material changes, except that the opacities in the vitreous grew less. On August 5th, one month after the accident, the ruptures could easily be seen, and their centers were very much whiter. Vision was 20-50. There was contraction of the fields for all colors. The patient then passed from under my observation and I have not seen him since.

Two points are of interest in this case:

First, the presence of multiple ruptures which are not quite so frequently seen, and the presence of these on the nasal side of the optic disk.

Second, the contraction of the fields of vision showed that there was also great *commotio retinæ* in addition to the choroidal lesion. Rupture of the choroid is by no means an infrequent occurrence after sharp blows upon the eye, but because of other attendant conditions, such as blood in the vitreous chamber, it is sometimes overlooked until some time after its occurrence.

Just why the choroid should rupture and in some instances the retina escape a similar condition is difficult to say, especially when we note how thin both of these tunics are and how closely adherent together they exist. Most usually when the choroid ruptures we have an involvement of the retina.

Such ruptures are most usually the result of sudden concussion upon the eyeball, such as blows from sticks, pieces of rock and mud, baseballs, the human fist, etc.

The exterior portions of the eye frequently show no changes whatever, unless there should be ecchymosis enough to produce the typical "black eye."

Following such an injury to the eyeball there may be extensive hemorrhages into the vitreous or even into the anterior chamber and thus obscure for some time the pathological condition of the choroid and retina. On the other hand, such accidents may cause a rupture without attendant hemorrhage, except possibly in the choroid itself. These ruptures are usually located around and nearby the optic disk, at the posterior pole of the eye. Some writers hold that they seldom occur on the nasal side, although such was the condition in the case reported by me.

The form the ruptures assume is usually semi-circular and rarely linear in character, corresponding seemingly to the contour of the disk. Horizontal ruptures are rare, as also the vertical ones, a case of the latter having been reported by deWecker as an anomaly where the vertical rupture occurred between the disk and the macular lutea. Ruptures occur single or multiple, in the latter case one usually being large and the others gradually tapering in size, but all being more or less in the same line.

After the hemorrhage, which usually occurs at the time of the accident, has been absorbed, these ruptures by the ophthalmoscope appear crescentic in shape, a yellowish or yellowish-white color frequently pigmented around their edges.

Such ruptures never close, becoming wider as time progresses, and sometimes producing decided nutritive changes in the tissues around them. The usually accepted explanation for these ruptures is that given by Becker, and that is that the optic nerve at the moment of the blow is driven into the sclerotic, producing concentrically to the disk a fold or series of folds in the choroid and retina, along which the former gives way. This certainly seems a rational explanation since we find these ruptures almost without an exception limited to the region around the optic disk.

As a rule there is little or no pain connected with this pathological condition, except perhaps during the first few days of the traumatism. Vision is interfered with the more the choroid and retina around the macula are involved.

The prognosis in these cases is unfavorable, but only so far as the vision is concerned. Knapp says the subsequent impairment of sight is the rule in these cases, and that although in the beginning the retina may not appear to be affected, later it is very apt to become involved.

Saemisch says that a return to normal acuity of vision is rare.

Treatment in these cases, just as treatment for many other pathologic conditions of the fundus, is of little avail.

During the first few days depletive measures and the internal administration of the iodides to aid in the absorption of blood, is all that can be done. The final result cannot be foretold and the ophthalmologist must trust to nature and the good Lord.

Grand Opera House Blk.

